# Analysis of Plant and Soil Restoration Process and Degree of Refuse Dumps in Open-Pit Coal Mining Areas

**DOI:** 10.3390/ijerph17061975

**Published:** 2020-03-17

**Authors:** Xinhui Li, Shaogang Lei, Feng Liu, Weizhong Wang

**Affiliations:** 1 Engineering Research Center of Ministry of Education for Mine Ecological Restoration, China University of Mining and Technology, Xuzhou 221116, China; xinhui.li@cumt.edu.cn; 2 School of Environment Science and Spatial Informatics, China University of Mining and Technology, Xuzhou 221116, China; 3 Environmental Restoration and Management Center of Jungar Banner Mining Area, Ordos 017100, China; 18647179995@163.com (F.L.); wvz123888@163.com (W.W.)

**Keywords:** plant community characteristics, soil properties, refuse dumps, restoration, ecological stability

## Abstract

Vegetation and soil restoration are the key to ecological reconstruction in the damaged areas of open-pit coal mining areas. Ecological stability is an important indicator of the degree of ecological restoration. In this study, the ecological stability and the process of plant and soil restoration were investigated at different refuse dumps in three coal mines, namely, the Wulanhada (WLHD) coal mine, the Liulingou (LLG) coal mine, and the Jinzhengtai (JZT) coal mine, in Jungar Banner. Results show that organic matter, total N, available N, and available K increased with the increase in restoration age at the two coal mines of WLHD and LLG. In the JZT coal mine, organic matter, total N, and available K firstly increased, and then slightly decreased with the increase in restoration age. The redundancy analysis indicates that most reclaimed mine soil properties (including soil moisture content, organic matter, total N, and available K) are positively correlated with plant species diversity in the three coal mines, while soil pH and soil bulk density showed a negative correlation with plant species diversity. Plant parameters increased with the years since revegetation, except the Pielou index for the WLHD coal mine, and the Pielou and Margalef indexes for the JZT coal mine. The Euclidean distance between the restoration areas and the natural reference areas decreased with the increase in restoration age. Our findings suggest that, in the three coal mines, the change law of ecological stability conformed to the logistic succession model. The same degree of ecological stability in different refuse dumps may correspond to different degrees of vegetation and soil development. This study emphasizes that ecological restoration in mining areas could benefit the structure of the plant community and the recovery of soil properties, which would eventually improve the ecological stability of coal mining areas.

## 1. Introduction

Seventy-five percent of the added value of coal production worldwide originates from opencast coal mining, and, in China, opencast coal production occupies 15% of the total coal production [1]. Opencast coal mine areas currently represent the most typically degraded ecosystem [2]. In recent years, the development of coal resources in China gradually moved toward the western semi-arid areas. The exploitation of coal mines caused a series of environmental problems, such as ground subsidence [3], vegetation degradation [4], soil erosion [5], and heavy metal pollution [6,7], with the largest impacts on vegetation and soil. Compared with natural reference areas, vegetation cover in coal mine areas is lower because of the direct removal of vegetation. Furthermore, the great change of topography in the mining process certainly affects the conditions of vegetation growth. Previous studies showed that differences in topography can lead to significant differences in light, heat, water, and soil nutrients [8,9], affecting the absorption of nutrients and water from plant roots. Water is the limiting factor in semi-arid areas, and it is directly involved in material transformation between soil and vegetation [10]. Moreover, it is a vital factor in determining ecological structure and function [11], especially in semi-arid aeolian areas. Another study uncovered that the divergence in soil nutrients influences plant species diversity [12]. Conversely, the feedback of vegetation through nutrient circulation has an effect on the availability of soil nutrients, as well as on its litter and root system. Thus, vegetation and soil damage in mining areas further results in imbalances of material cycle and energy flow [13], breaking their ecological structure and function [14,15], making the ecosystem lose the ability to resist external interference, and ultimately leading to the ecological instability of the mining area [16,17].

The concept of ecological stability (ES) originates from that of community stability [18]. ES is commonly defined as the ability of an ecosystem to resist changes in the presence of perturbations [19]. As vegetation and soil restoration can ensure that damaged habitats restore their ability of resistance [17], mining areas can constantly move inside a virtuous circle and maintain a relatively stable state, i.e., ecological stability [20]. Therefore, ES can be used as an important indicator of ecological restoration. The purpose of ecological restoration in open-pit coal mining areas is not only to study the relationship between vegetation and soil restoration during the restoration process, but, more importantly, to study whether these areas recovered to, or even exceeded their pre-damaged levels. Thus, assessing the success of ecological restoration projects is critical to justify the use of restoration in natural resource management, and to improve best practices in open-pit coal mine areas [21]. Until now, most studies focused only on the law of vegetation restoration [10], the improvement of soil properties [22], or on the interaction between vegetation and soil restoration [23,24,25,26]. Soil organic matter and total nitrogen are key indicators in the process of vegetation growth [27], although their content is generally lower in the soil of mining areas [28]. However, a previous study showed that vegetation richness, as well as organic C and total N and P accumulated in soil, increased with rehabilitation ages [23]. Wang [29] discovered that both the cumulative law of soil properties and the plant biomass were in line with the logistic growth model, with the increase of recovery age. Moreover, the chemical properties of surface soil were better than those of the lower soil with the increase of recovery age. During the ecological restoration process, soil properties can exceed those of natural areas. It was found that the content of available K (AK) and available P (AP) in the soil after eight years of reclamation was higher than in natural areas [30]. Moreover, a high sensitivity of vegetation to environmental factors and species composition was discovered in restoration areas, by comparison with natural areas [31]. Another study explained that an excess of nutrients (overfertilization) may be phytotoxic [32].

There are currently few studies about the ES of mining areas after restoration. Studies were performed to assess vegetation stability using plant diversity [33,34] or normalized difference vegetation index (NDVI) time series [35]. Considering that vegetation and soil restoration are equally important and have a complex interaction [26], and that large-scale research methods are not applicable to coal mining restoration areas, we introduced a coupling coordination model to reflect the ES of restoration areas. The coupling coordination model refers to the concept of capacitive coupling and capacitive coupling coefficient model in physics [36]. In physics, coupling refers to a phenomenon where two (or more) systems or motion forms affect each other through a variety of interactions. Coordination is a benign correlation between two (or more) systems or system elements, which guarantee the stable development of multiple systems or elements [37]. When the system or the internal elements of the system cooperate properly and complement each other, there is a benign coupling; otherwise, there is a malignant coupling. Coordination reflects a trend of the system from disorder to order in the development process between two (or more) systems, or between the internal elements of the system [38]. System entropy theory points out that order is stability [39]. With the passage of time, vegetation and soil properties gradually develop from a disordered state into an ordered state in coal mining restoration areas, which recover and stabilize with a reasonable structure and a perfect function [40,41,42]. Therefore, it is feasible to study the stability of mining areas by using the coupling coordination model [43]. At present, the coupling coordination model is mainly used to solve the relationship between economy, tourism, ecological environment, cities, and other macro aspects, while few studies were conducted on coal mining restoration areas [44,45].

In recent years, massive ecological restoration projects were implemented in the open-pit coal mining areas of Jungar Banner. Based on the space-for-time substitution approach, the restoration process and the degree of vegetation and soil were analyzed in three coal mines with different restoration years. This study aims to (1) characterize the change law of plant communities and soil properties with restoration years in refuse dumps, (2) characterize the interaction between plant community characteristics and reclaimed soil properties, and (3) analyze the ES of refuse dumps in open-pit coal mines. The findings of this study can shed light on the ecological stability of the restoration ecosystem, and on the management strategies for the restoration of refuse dumps.

## 2. Materials and Methods

### 2.1. The Study Area

**Figure 1:**
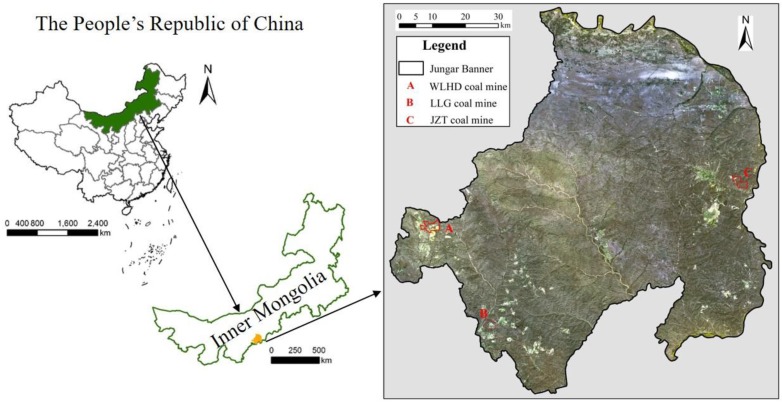
Location of the study area. A, B, and C represent the WLHD coal mine, the LLG coal mine, and the JZT coal mine, respectively.

The study area extends for 7.55 × 10^3^ km^2^; it is located in Jungar Banner (39°16′–40°20′ north (N), 110°05′–111°27′ east (E); 1100–1250 m above sea level (a.s.l.)), in the eastern part of the Inner Mongolia Autonomous Region, China. The topography is high in the northwest and low in the southeast. The main topographic features include arsenic sandstone, aeolian sand, and loess gully. The mineral resources are distributed mainly in the western, southwestern, and eastern parts of Jungar Banner, with barren land and a fragile ecological environment. The study area is characterized by a semi-arid climate with uneven precipitation distribution, mainly concentrated in the summer. The average annual precipitation is 408 mm, while the average annual evaporation capacity is about 2100 mm. The annual average temperature is 7.2 °C. The Yellow River is the largest surface water body in Jungar Banner. It is not only a regional discharge channel of surface water and groundwater, but also the supply source of groundwater in this area. Jungar Banner is a national comprehensive energy base, characterized by a high-intensity, large-scale coal exploitation. We selected a mine in each mine group for field experiments (A: WLHD coal mine, B: LLG coal mine, C: JZT coal mine; Figure 1).

### 2.2. Experimental Design and Method

#### 2.2.1. Sampling

On the basis of extensive data collection on the background conditions, reclamation time, and scope of coal mining, we selected the restoration areas (RA) with different years and natural reference areas (NA) in different typical coal mining areas. To select contrast areas, the NA was close to RA. The main reclamation vegetation in the study area is composed of grasses (*Medicago sativa* L., *Melilotus officinalis* (L.) Pall., *Astragalus propinquus* Schischkin., *Elymus dahuricus* Turcz.), sub-shrubs (*Artemisia desertorum* Spreng. Syst. Veg.), and shrubs (*Hippophae rhamnoides* Linn., *Caragana korshinskii* Kom.). At each of the restoration areas and the natural reference areas, three plots (in the WLHD and LLG coal mines) or two plots (in JZT coal mine) were randomly chosen and covered (herbs for 1 m × 1 m, shrubs for 2 m × 2 m). The position of the sampling sites was recorded using a portable global position system GPS (eTrex Venture, Garmin, Lenexa, KS, USA). Soil sampling and vegetation survey were performed in August 2018 (Table 1).

#### 2.2.2. Method

Plant surveys were conducted in each plot. All the plants in a plot were recorded and identified at the species level, according to Chang and Liu [46]. We recorded the plant species (SP), the total number of plants (NP), and the terrain and environmental characteristics provided by the Environmental Restoration and Management Center of the Jungar Banner mining area. We used a combination of parameters representing plant diversity, such as the Shannon–Wiener index (H’) to reflect relative abundances, the Simpson index (D) to measure dominance, the Pielou index (J) to express evenness, and the Margalef index (Mg) to reflect richness. These methods were described according to Tsafack [47]. Prior to sampling, the litter layer was removed, soil samples were collected at a depth between 5 and 30 cm, and each sample was composed of four subsamples from the center point and the surrounding area. Immediately after collection, the samples were mixed and placed in an aseptic bag (about 50 g). Soil moisture content (SMC) was measured in the field using an ML3X soil moisture tester (Delta-T, Inc., UK), with three replicates at each sampling site [48]. Soil bulk density (BD) was measured using the ring-cut method (cutting ring size: diameter 50.46 mm, height 50 mm). Except for soil moisture content and soil bulk density, all soil chemical properties were measured in the laboratory. Total salt content (TS) was measured through the residue drying–quality method. Soil organic matter (SOM) was measured using the method of potassium dichromate; soil pH was measured with a pH meter (Origin Research Inc.); total N was determined with the salicylate–hypochlorite method, using a SANplus Segmented Flow Autoanalyzer (SANplus) after semi-micro-Kjeldahl [23]; available N and P were determined using the alkaline hydrolyzation diffusion method and the 0.5 mol/L NaHCO3 extraction-Mo-Sb colorimetric method, respectively. NH4OAC extraction–flame photometry was used to detect available K [49]. All methods followed those described previously [50].

### 2.3. Data Analysis

#### 2.3.1. Significant Difference Analysis

One-way analysis of variance (ANOVA) followed by Tukey’s honestly significant difference (HSD) test at *p* < 0.05 was used to determine any significant difference between means of plant community characteristics and soil properties in different restoration areas and natural reference areas. Data analysis was carried out using SPSS 19.0 for Windows (SPSS Inc., Chicago, USA).

#### 2.3.2. Multivariate Statistical Analysis

Multivariate statistical analysis was performed to explore the relationship among plant community characteristics, soil properties, and sample plots using the Canoco 5. Software (Center for Biometry, Wageningen, the Netherlands). To determine the best analytical model (i.e., the linear model or the unimodal model), the species data were analyzed through a detrended corresponding analysis (DCA) prior to the analysis. Canoco 5 (through its Canoco Adviser) can suggest the choice of the appropriate analysis model [51]. In this study, the statistical significance (at the 5% level) of relationships between plant community characteristics and soil properties was assessed using the Monte Carlo test on 499 random permutations to test the null hypothesis that the plant community was unrelated to environmental variables [52,53].

#### 2.3.3. Ecological Stability Analysis

To calculate the ES of restoration areas, we considered that the RA and NA consist only of the plant and soil systems. Firstly, the comprehensive evaluation indexes of the plant community and soil properties were constructed in the model evaluation (Equation (1) and (2)), which revealed the qualities of plant community and soil properties. Furthermore, we calculated the ratio of f(x) and f(y) to represent the degrees of vegetation and soil development.

(1) $$f\left(x\right)=\overset{m}{\underset{i=1}{\sum }}a_{i}x_{i}$$

(2) $$f\left(y\right)=\overset{n}{\underset{j=i}{\sum }}b_{j}y_{j}$$

where *i* and *j* represent the number of plant community characteristics and soil properties, respectively; *a_i_* and *b_i_* represent the corresponding weight value of plant community characteristics and soil properties, respectively; *x_i_* and *y_j_* represent the standardized value of each indicator.

Secondly, we constructed a coupling index of plant community characteristics and soil properties to assess their composite state (Equation (3)). Finally, by introducing the index overall development level of the system (Equation (4)), we calculated the ES (Equation (5)) including both the plant and the soil systems. The plant and soil indicators were standardized by using the extreme value method, and the entropy weight method was used to determine the relative weight of each indicator.

(3) $$C=\sqrt{\frac{(f\left(x\right)*f(y))}{{\left(\frac{f\left(x\right)+f(y)}{2}\right)}^{2}}}$$

(4) T=αf(x)+βf(y)

(5) $$D=\sqrt{C*T}$$

where *C* is the coupling index of plant community characteristics and soil properties; *T* is the overall development level of the system; *D* is the coupling coordination index of plant community characteristics and soil properties, reflecting ES; α and β are the contribution rates of soil and vegetation, respectively. As vegetation and soil restoration are equally important to ES, both α and β were set as 0.5. The *D* value was set in the range of 0–1, with a higher value indicating a higher regional ES, and vice versa. Following Peng et al. [54], we divided ES into five categories (Table 2).

## 3. Results

### 3.1. Soil Properities

**Figure 2:**
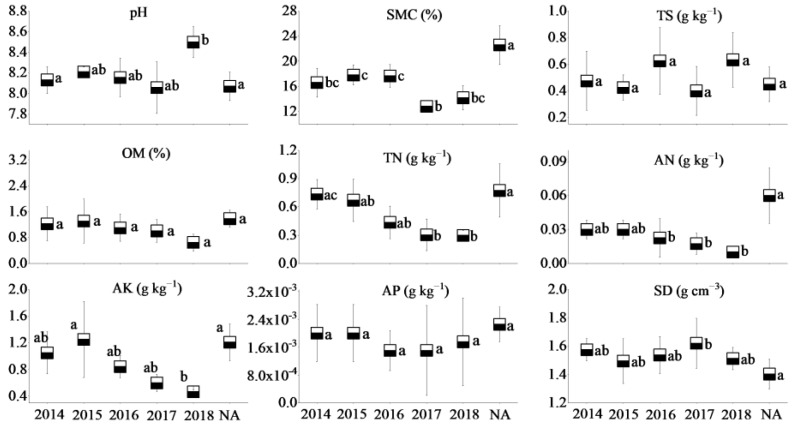
Soil properties of different restoration ages and natural reference areas in the WLHD coal mine (mean values followed by the same letter within a column are not significantly different at the *p* = 0.05 level; mean values followed by different letters within a column are significantly different at the *p* < 0.05 level, according to Tukey’s honestly significant difference (HSD) test).

**Figure 3:**
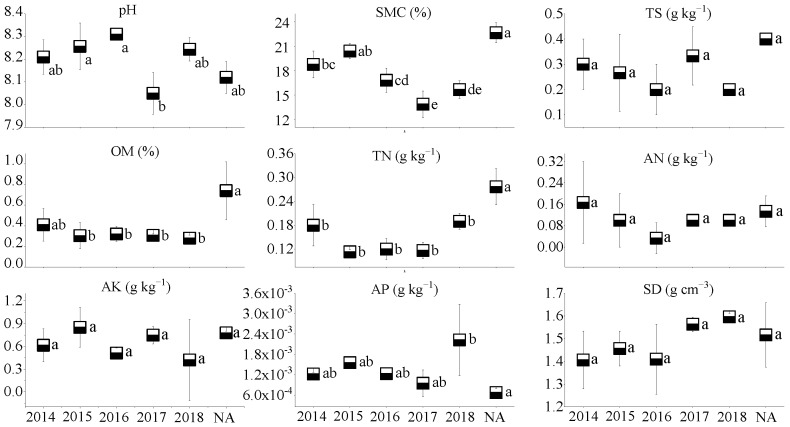
Soil properties of different restoration ages and natural reference areas in the LLG coal mine (mean values followed by the same letter within a column are not significantly different at the *p* = 0.05 level; mean values followed by different letters within a column are significantly different at the *p* < 0.05 level, according to Tukey’s HSD test).

**Figure 4:**
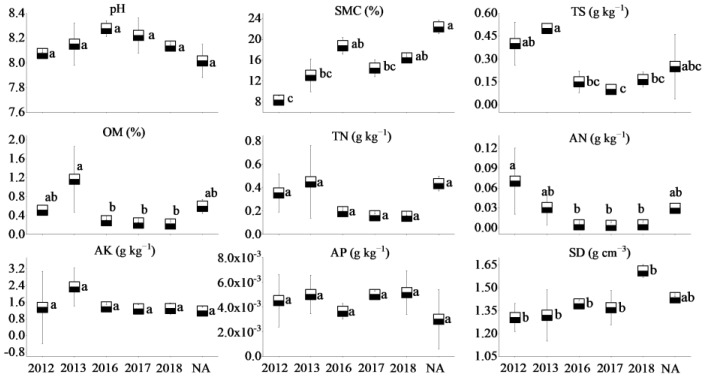
Soil properties of different restoration ages and natural reference areas in the JZT coal mine (mean values followed by the same letter within a column are not significantly different at the *p* = 0.05 level; mean values followed by different letters within a column are significantly different at the *p* < 0.05 level, according to Tukey’s HSD test).

The soil of the three coal mines is alkaline, with soil pH values ranging from 8.05 to 8.31. Soil moisture content, total N, and available N in the NA of WLHD coal mine were higher (*p* < 0.05) than those of different RAs. Significant differences were found in total N and available K between the early revegetated sites (RA14 and RA15) and the later groups (RA18; *p* < 0.05). However, no significant differences in total salt, organic matter, and available P were observed across different years (*p* > 0.05; Figure 2). Furthermore, the natural reference site of the LLG coal mine had a higher (*p* < 0.05) soil moisture content, organic matter, and total N. In general, no significant differences in total salt, organic matter, total N, and available nutrients were found across different restoration years (Figure 3). Soil moisture content in the NA of the JZT coal mine was higher (*p* < 0.05) than in different restoration sites (*p* < 0.05). Significant differences were found in total salt, organic matter, and available N between the early revegetated sites (RA12 and RA13) and the later groups (RA17 and RA18; *p* < 0.05). However, no significant differences in soil pH, total N, available K, and available P were observed across different years (*p* > 0.05). Moreover, RA12 and RA13 in the JZT coal mine had the highest organic matter and available N, respectively (Figure 4). In relation to bulk density, no significant differences were observed among the different sites in the three coal mines (*p* > 0.05), although the bulk density decreased after vegetation restoration. In the WLHD and LLG coal mines, organic matter, total N, available N, and available K increased with the increase in restoration age. In the JZT coal mine, organic matter, total N, and available K firstly increased and then decreased with the increase in restoration age.

In the three coal mines, the soil moisture content at the newly rehabilitated area (RA18) was 60–75% of the value at the NA. The organic matter content in the newly rehabilitated area (RA18) was less than 50% of that in the NA. Additionally, apart from the LLG coal mine, total N and available N (except in LLG coal mine) in the newly rehabilitated area (RA18) were less than 40% and 20% of the value in the NA, respectively. Over time, the gap between the values of soil properties will gradually narrow.

### 3.2. Plant Community Characteristics

**Figure 5:**
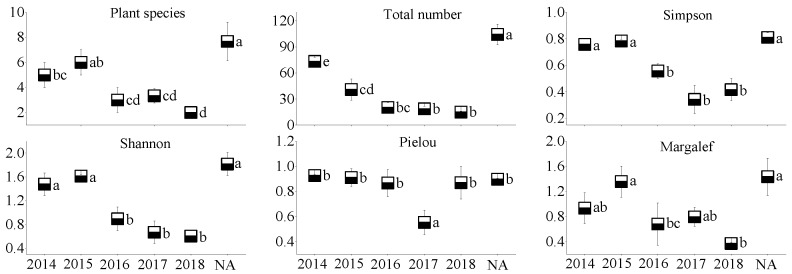
Plant community characteristics of different restoration ages and natural reference areas in the WLHD coal mine (mean values followed by the same letter within a column are not significantly different at the *p* = 0.05 level; mean values followed by different letters within a column are significantly different at the *p* < 0.05 level, according to Tukey’s HSD test).

**Figure 6:**
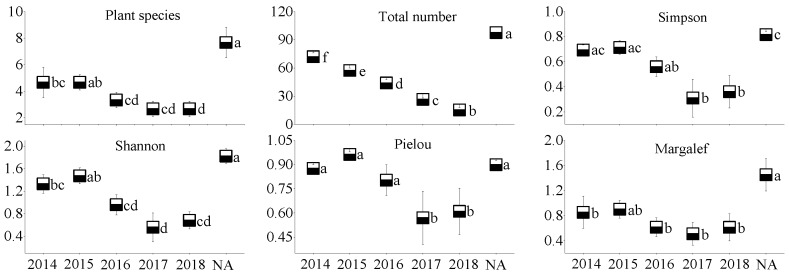
Plant community characteristics of different restoration ages and natural reference areas in the LLG coal mine (mean values followed by the same letter within a column are not significantly different at the *p* = 0.05 level; mean values followed by different letters within a column are significantly different at the *p* < 0.05 level, according to Tukey’s HSD test).

**Figure 7:**
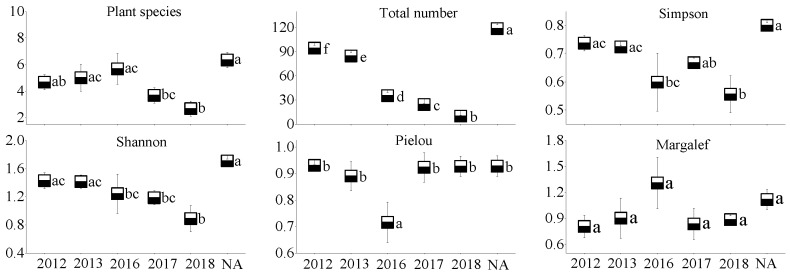
Plant community characteristics of different restoration ages and natural reference areas in the JZT coal mine (mean values followed by the same letter within a column are not significantly different at the *p* = 0.05 level; mean values followed by different letters within a column are significantly different at the *p* < 0.05 level, according to Tukey’s HSD test).

In the WLHD coal mine (Figure 5), an increase in plant species, the Pielou index, and the Margalef index occurred with the restoration time; however, no significant differences were observed among the different restoration areas (*p* > 0.05). In contrast, significant differences were found in the Simpson index and in the Shannon index between the early revegetated sites (RA14 and RA15) and the later groups (RA16, RA17, and RA18; *p* < 0.05). Likewise, significant differences were found in the Simpson index, the Shannon index, and the Pielou index between the early revegetated sites (RA14 and RA15; Figure 6) and the later groups in the LLG coal mine (RA17 and RA18; *p* < 0.05). In the JZT coal mine (Figure 7), not all parameters increased as the time of restoration increased. No significant differences were observed in plant species, the Pielou index, or in the Margalef index (*p* > 0.05); however, significant differences were observed in the Simpson index and in the Shannon index between the early revegetated sites (RA12 and RA13) and the later site (RA18; *p* < 0.05).

Moreover, all the plant community parameters in the NA of the three coal mines were the highest, except for the Pielou index in the three coal mines and for the Margalef index in the JZT coal mine. Basically, all the parameters, except the Pielou index in the newly rehabilitated area (RA18), were less than 50% of those in the NA. Over time, the gap between parameter values will gradually narrow.

### 3.3. RDA of Plant Community and Soil Properties

**Figure 8:**
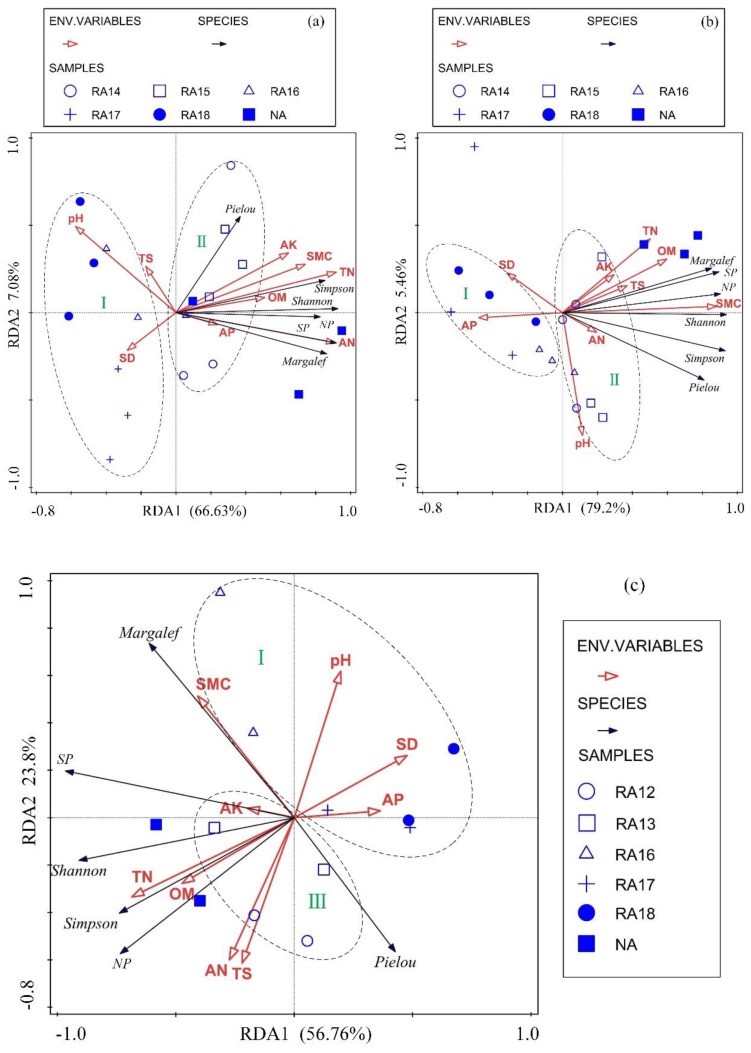
Triplot of the first two redundancy analysis (RDA) axes of plant community characteristics, soil properties, and plots. Black arrows indicate the plant parameters, while red arrows indicate the soil parameters. SMC: soil moisture content; TS: total salt content; OM: organic matter; TN: total nitrogen; AN: available nitrogen; AK: available potassium; AP: available phosphorus; SD: soil bulk density; SP: plant species; NP: total number of plants; (**a**) WLHD coal mine; (**b**) LLG coal mine; (**c**) JZT coal mine.

The detrended correspondence analysis (DCA) suggested that the redundancy analysis (RDA) was the best analytical model for this study. The results of the RDA show that, in the WLHD coal mine, 77.7% of the variance of plant community characteristics could be explained by the soil properties, from the canonical sum of the eigenvalues (Figure 8a). Not every soil property had a significant influence on plant community. The Monte Carlo permutation test showed that significant soil parameters included total nitrogen (TN; *p* = 0.002, F-value = 20.9) and available nitrogen (AN; *p* = 0.022, F-value = 3.8), and their impact on the plant community accounted for 65.5% of all soil properties. All other soil properties, except for soil bulk density (SD), TS, and soil pH, were positively correlated with plant community characteristics. The positive correlation indicators were clustered around TN, organic matter (OM), and SMC, indicating a strong correlation between these soil parameters.

The results of the RDA show that, in the LLG coal mines, 86.3% of the variance of plant community characteristics could be explained by the soil properties (Figure 8b). The Monte Carlo permutation test indicated that SMC (*p* = 0.002, F-value = 25.6) and soil pH (*p* = 0.044, F-value = 3.7) had a significant effect on plant community, and their impact on plant community accounted for 61.6% and 6.1% of all soil properties, respectively. SMC, OM, TN, AN, available potassium (AK), and TS showed a positive correlation with plant community characteristics; among these, SMC and OM had the strongest correlation with plant community.

The results of the RDA indicate that, in the JZT coal mine, 84.2% of the variance of plant community characteristics could be explained by soil properties (Figure 8c). The Monte Carlo permutation test indicated that TN (*p* = 0.024, F-value = 4.2) had a significant effect on plant community, and its influence on plant community accounted for 29.5% of all soil properties. Furthermore, the effects of SMC on plant community accounted for 14.4% of all soil properties. However, SMC (*p* = 0.108, F-value = 2.3) had no significant influence on plant community. SMC, OM, TN, and AK were positively correlated with plant community characteristics (except for the Pielou index). In addition, the RDA results also revealed that the Euclidean distance between RA and NA decreased (from stage I to stage III) with the increase in restoration age.

### 3.4. Ecological Stability Analysis after Restoration in the Mining Area

**Figure 9:**
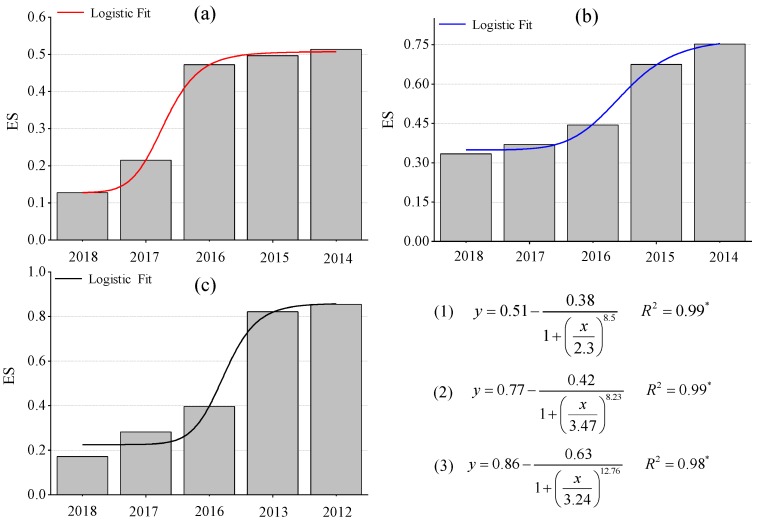
Change law of ecological stability of the refuse dumps in different coal mine areas across different restoration ages; *y* represents the value of ES and *x* represents the restoration time; equation (**1**) represents the logistic succession model of ecological stability in WLHD coal mine, equation (**2**) represents the logistic succession model of ecological stability in LLG coal mine, and equation (**3**) represents the logistic succession model of ecological stability in JZT coal mine (* *p* < 0.05, (**a**) WLHD coal mine; (**b**) LLG coal mine; (**c**) JZT coal mine).

Table 3 and Figure 9 clearly show that the ES in the restoration sites of the three coal mines increased with the increase in restoration age, which conforms to the logistic succession model. The ES of the WLHD and LLG coal mines was described as follows: NA > RA14 > RA15 > RA16 > RA17 > RA18. Clearly, the level of ES in the investigated restoration sites of those two coal mines was lower than in the NA. However, the ES of the JZT coal mine was described as follows: RA12 > RA13 > NA > RA16 > RA17 > RA18, and the level of ES in the RA12 and RA13 exceeded that in the NA. The present results also show that, in the same restoration year, the vegetation and soil development degree varied across the refuse dumps of different coal mines. Even if the ecological stability of different refuse dumps is the same, the corresponding vegetation and soil development degree may be diverse.

## 4. Discussion

### 4.1. Effects of Mining and Restoration on Soil Properties and Plant Community Characteristics

Mining activities affect the composition of plant communities and soil properties in mining areas, inevitably leading to the degradation of vegetation and the loss of soil properties [55,56]. In accordance with previous studies [28], most of the soil nutrients measured in this study increased with the increase in restoration age (Figure 2, Figure 3 and Figure 4), suggesting that a revegetative approach is effective to enhance topsoil recovery in refuse dumps. In general, plants can affect the soil nutrients [57], which may be attributed to the conversion from leaf litter fall to humus in the topsoil [30]. Thus, soil nutrients were relatively lower in the newly RA, as the seedlings and soil were in the initial stage of mutual feedback. Compared with the SMC of other restoration sites, it was found that the SMC of newly reclaimed soil (RA18) was not the lowest because of the artificial irrigation after restoration. Over time, the water retention ability gradually increased, owing to improvements in soil structure [58]. Because of the frequent movements of heavy machinery excavating and replacing topsoil material in the restoration process, the reclaimed mine soils are highly compact, leading to high soil bulk density. In agreement with a previous study [59], it was found that soil bulk density in the RAs of the three coal mines was higher than in the NA. Moreover, soil bulk density was found to decrease in response to time since restoration, indicating a recovery from soil compaction and improved soil structure [60]. Soil pH plays an important role in ecological restoration owing to its function in moderating plant nutrients availability. Soil pH values in the RAs were found to be higher than in the NA, as severe soil disturbance inevitably causes changes in soil pH, usually resulting in soil pH increase [61]. Other studies found that soil acidification was also attributed to the process of organic matter accumulation [23,62,63]. In our study, soil pH was alkaline in the three coal mines and the phenomenon of acidification with the accumulation of organic matter was not clear. The results of this study indicate that vegetation restoration clearly enhanced the plant species, the total number of plants, and plant species diversity in the refuse dumps.

### 4.2. Interaction between Plant Community Characteristics and Soil Properties during the Restoration Process

The interaction between plant community characteristics and reclaimed soil properties was complex. It was found that SD had a negative correlation with plant diversity indexes, which was in line with the findings of Lu [64]. This could be attributed to the fact that the increase in soil density made the soil have less water-holding capacity, and the resulting water deficit inhibited the plant growth. Soil pH is regarded as the main factor regulating species diversity [58]; in our case study, it was also an important factor controlling plant community characteristics. A positive correlation between soil pH and plant diversity appeared in the LLG coal mine, while the opposite result was found in the WLHD and JZT coal mines. The reason for this difference is that soil pH values exceeding the plant growth range can block the plant growth and decrease their diversity indexes. The plant diversity indexes were positively correlated with nutrients and SMC. According to the results of the RDA analysis, these indexes were clustered around the SMC. This is because SMC is a key factor in promoting plant growth in arid areas [65]; conversely, plant roots play an important role in improving soil water storage and conservation capacity [48]. With the succession of the plant community, increasing plant diversity had positive effects on organic matter accumulation via continuous litter production and root decay [23]. In addition, artificial restoration plants can change soil micro-environmental conditions, promoting microbial activity such as nitrogen-fixing bacteria. This is in an agreement with Reference [26], who concluded that total N and available N positively correlate with plant diversity indexes. Additionally, the results of the RDA show that the restoration process was divided into three stages, from stage I to stage III (Figure 8). It is known that the predicted increase of samples occurs in the direction indicated by the arrow. In the initial stage of restoration, the samples of newly rehabilitated sites (RA18, RA17, and RA16 in this study) were located in the opposite direction of the arrow, except for the arrow of soil pH and SD. In the second stage of restoration, the samples of RA14 and RA15 were in the middle of the arrow. In the third stage of restoration, the samples of RA13 and RA12 were near the top of the arrow, and the Euclidean distance between the restoration sites (RA12 and RA13) and the NA was decreasing. It is confirmed that the ecological restoration of the mining areas gradually achieved a recovery corresponding to the pre-mining ecosystem. However, as shown by the results of this study, it is not possible to predict how long it will take to recover to the pre-mining state.

### 4.3. The Change Law of Ecological Stability under Restoration Activities

Soil restoration and vegetation restoration are equally important in ecological restoration activities in semi-arid coal mines [26]. The purpose of ecological restoration in open-pit coal mining areas is to recover to pre-mining level, reaching a level of ES that is comparable to the NA [26,45]. Figure 9 indicates that ES rose in the three coal mines with the increase in restoration age, which conforms to the logistic succession model. It was found that the correlation coefficients of each model were relatively high, illustrating that the model could better reflect the change law and recovery degree of ES in the refuse dumps of the three coal mines.

We can clearly see that the ecological stability and the development of plant soil types did not follow a one-to-one mapping. Even if the ES of different refuse dumps was the same, the corresponding vegetation and soil development degree was different. The ES of RA14, RA15, and RA16 in the WLHD coal mine was sub-stable. Meanwhile, the vegetation and soil development degree of RA14 was VSD; however, the vegetation and soil development degree of RA15, RA16, RA17, and RA18 was SL. This indicates that soil condition in the WLHD coal mine is poor. Long-term mining disturbances exacerbated water and wind erosion, resulting in the staggered distribution of chestnut, skeleton, and aeolian sandy soils in the WLHD coal mine. Thus, the soil in the WLHD coal mine has thin humic substances and poor fertility, which indicates the need to strengthen soil management and increase fertilization. According to the results of the RDA, both total N and available N exerted a significant impact on plant communities in the WLHD coal mine, which inspired the rational addition of nitrogen fertilizers in the process of fertilization. A previous study [23] showed that organic matter accumulated via continuous litter production and root decay after vegetation restoration. Therefore, with the increase in restoration age, the vegetation and soil development degree in RA14 gradually synchronized in accordance with the NA. Even then, it was clear that the ES of RA14 was far from that of NA. In the LLG coal mine, the vegetation and soil development degree of RA14, RA15, and RA16 were found to belong to VL, despite the improvement of the ES. Having knowledge of the plant composition in restoration areas, the main plant composition in the early stage of restoration was supposed to be grasses, in light of enhancing soil fertility [10]. With the increase in restoration age, the grass would deteriorate; thus, the vegetation and soil development degree of RA14, RA15, and RA16 would become PL, indicating that soil fertility was not fully used, and that the vegetation sparse areas could be properly replanted [45]. A combination of grasses and shrubs was more suitable to provide long-term environmental benefits and increase the degree of the ES. The NA, although undamaged, was of the PL type, which indicates that more irrigation water was needed. In the JZT coal mine, sufficient quantities of nitrogen fertilizers need to be allocated appropriately. The ES of RA12, RA13, and NA in the JZT coal mine was stable, and the corresponding vegetation and soil development degrees were PSS, PSS, and PL, respectively. Apparently, the growth of vegetation in RA12 and RA13 was superior to that in NA. This result is consistent with the conclusion that an appropriate artificial disturbance could improve the relationship between plant and soil, even surpassing the plant and soil condition of undamaged areas [28].

Considering the relatively short restoration age of the refuse dumps selected, future studies should concentrate on the areas that were reclaimed for more than 10 or 20 years.

## 5. Conclusions

Mining and restoration activities significantly altered the plant community characteristics, soil properties, and ecological stability. Restoration activities performed at the refuse dumps of coal mines improved the soil environment for the colonization and establishment of plant species. The concentrations of OM, total N, available N, and available K increased with the years since restoration in both the WLHD and the LLG coal mines. In parallel, the concentrations of OM, total N, and available K increased at first, and then slightly decreased with the increase in restoration ages. Plant parameters increased with the years since revegetation, except the Pielou index for the WLHD coal mine, and both the Pielou and the Margalef indexes for the JZT coal mine. The results from the RDA analysis showed that plant community characteristics and soil properties are closely correlated, and that changes in soil properties may have an effect on plant community characteristics. The results indicate that total N and available N in the WLHD coal mine, SMC and soil pH in the LLG coal mine, and total N in the JZT coal mine have a significant influence on plant community characteristics. Those factors are regarded as key drivers of the future structure of plant community and ecological stability. Moreover, our findings suggest that ecological restoration can improve ecological stability. Over time, it is possible to gradually achieve the recovery to, or even exceed the corresponding pre-mining state.

## Figures and Tables

**Table 1:** Basic conditions of the natural reference areas and the refuse dumps with different restoration ages in various coal mines.

Site	Location	Topography	Soil Type	Restoration Age	
WLHD Coal mine	111°13′00″– 110°17′05″ east (E), 39°42′00″– 39°44′00″ north (N)	Highland erosion hilly topography	Chestnut and skeleton soils	NA	Grass, sub-shrub, shrub
				RA14	Grass, sub-shrub, shrub
				RA15	Grass, sub-shrub
				RA16	Grass, sub-shrub
				RA17	Grass
				RA18	Grass
LLG Coal mine	110°26′25″– 110°29′09″ E 39°26′28″– 39°27′59″ N	Highland erosion hilly topography	Cinnamon soil	NA	Grass, sub-shrub, shrub
				RA14	Grass, sub-shrub
				RA15	Grass, sub-shrub
				RA16	Grass
				RA17	Grass
				RA18	Grass
JZT Coal mine	110°19′21″– 111°22′17″ E 39°49′06″– 39°51′27″ N	Low hilly and valley topography	Cinnamon and aeolian sandy soils	NA	Grass, sub-shrub, shrub
				RA12	Grass, sub-shrub, shrub
				RA13	Grass, sub-shrub
				RA16	Grass, sub-shrub
				RA17	Grass
				RA18	Grass

Note: RA12 means the areas reclaimed in 2012 a; RA13 means the areas reclaimed in 2013 a; RA14 means the areas reclaimed in 2014 a; RA15 means the areas reclaimed in 2015 a; RA16 means the areas reclaimed in 2016 a; RA17 means the areas reclaimed in 2017 a; RA18 means the areas reclaimed in 2018 a; NA means the natural reference area.

**Table 2:** Categories and standards of ecological stability and the vegetation and soil development degree.

D	Categories (Ecological Stability)	f(x)/f(y)	Sub-Categories (Vegetation and Soil Development Degree)
0 < D ≤ 0.2	Extremely unstable	f(x)/f(y) > 1.2	VL
		0.8 ≤ f(x)/f(y) ≤ 1.2	VSL
		f(x)/f(y) < 0.8	SL
0.2 < D ≤ 0.4	Unstable	f(x)/f(y) > 1.2	VL
		0.8 ≤ f(x)/f(y) ≤ 1.2	VSL
		f(x)/f(y) < 0.8	SL
0.4 < D ≤ 0.6	Sub-stable	f(x)/f(y) > 1.2	VL
		0.8 ≤ f(x)/f(y) ≤ 1.2	VSD
		f(x)/f(y) < 0.8	SL
0.6 < D ≤ 0.8	Nearly stable	f(x)/f(y) > 1.2	VL
		0.8 ≤ f(x)/f(y) ≤ 1.2	VSD
		f(x)/f(y) < 0.8	SL
0.8 < D ≤ 1.0	Stable	f(x)/f(y) > 1.2	VL
		0.8 ≤ f(x)/f(y) ≤ 1.2	VSD
		f(x)/f(y) < 0.8	SL

Note: D represents the threshold of ecological stability; VL represents the lagging type of vegetation restoration; SL represents the lagging type of soil restoration; VSL represents the synchronous lagging type of vegetation soil restoration; VSD represents the synchronous development type of vegetation soil restoration.

**Table 3:** Results of ecological stability and vegetation and soil development degree across the different restoration ages and natural reference areas.

	Restoration Age	D	Ecological Stability	f(x)/f(y)	Vegetation and Soil Development Degree
WLHD coal mine	RA14	0.51	Sub-stable	0.93	VSD
	RA15	0.49	Sub-stable	0.64	SL
	RA16	0.47	Sub-stable	0.57	SL
	RA17	0.22	Unstable	0.67	SL
	RA18	0.13	Extremely unstable	0.21	SL
	NA	0.63	Nearly stable	0.95	VSD
LLG coal mine	RA14	0.75	Nearly stable	1.17	VL
	RA15	0.67	Nearly stable	1.49	VL
	RA16	0.44	Sub-stable	2.48	VL
	RA17	0.37	Unstable	0.25	SL
	RA18	0.33	Unstable	0.11	SL
	NA	0.90	Stable	1.34	VL
JZT coal mine	RA12	0.85	Stable	1.02	VSD
	RA13	0.82	Stable	0.98	VSD
	RA16	0.40	Unstable	3.60	VL
	RA17	0.28	Unstable	3.92	VL
	RA18	0.17	Extremely unstable	0.14	SL
	NA	0.81	Stable	2.22	VL

*Note*. See Table 2 for an explanation of the abbreviations.
